# Single-Step BLUP with Varying Genotyping Effort in Open-Pollinated *Picea glauca*

**DOI:** 10.1534/g3.116.037895

**Published:** 2017-01-24

**Authors:** Blaise Ratcliffe, Omnia Gamal El-Dien, Eduardo P. Cappa, Ilga Porth, Jaroslav Klápště, Charles Chen, Yousry A. El-Kassaby

**Affiliations:** * Department of Forest and Conservation Sciences, Faculty of Forestry, University of British Columbia, Vancouver, British Columbia V6T 1Z4, Canada; † Pharmacognosy Department, Faculty of Pharmacy, Alexandria University, Alexandria, 21521, Egypt; ‡ Instituto Nacional de Tecnología Agropecuaria (INTA), Instituto de Recursos Biológicos, Las Cabañas y De Los Reseros s/n, 1686 Hurlingham, Buenos Aires, Argentina; § Départment des Sciences du Bois et de la Forêt, Faculté de Foresterie, de Géographie et Géomatique, Université Laval Québec, G1V 0A6, Canada; ** Department of Genetics and Physiology of Forest Trees, Faculty of Forestry and Wood Sciences, Czech University of Life Sciences Prague, 165 21 Praha 6, Czech Republic; †† Scion (New Zealand Forest Research Institute Ltd.), Whakarewarewa, Rotorua 3046, New Zealand; ‡‡ Department of Biochemistry and Molecular Biology, Oklahoma State University, Stillwater, Oklahoma 74078-3035

**Keywords:** bivariate mixed model, HBLUP, single-step BLUP, tree improvement, GenPred, Shared Data Resources, genomic selection

## Abstract

Maximization of genetic gain in forest tree breeding programs is contingent on the accuracy of the predicted breeding values and precision of the estimated genetic parameters. We investigated the effect of the combined use of contemporary pedigree information and genomic relatedness estimates on the accuracy of predicted breeding values and precision of estimated genetic parameters, as well as rankings of selection candidates, using single-step genomic evaluation (HBLUP). In this study, two traits with diverse heritabilities [tree height (HT) and wood density (WD)] were assessed at various levels of family genotyping efforts (0, 25, 50, 75, and 100%) from a population of white spruce (*Picea glauca*) consisting of 1694 trees from 214 open-pollinated families, representing 43 provenances in Québec, Canada. The results revealed that HBLUP bivariate analysis is effective in reducing the known bias in heritability estimates of open-pollinated populations, as it exposes hidden relatedness, potential pedigree errors, and inbreeding. The addition of genomic information in the analysis considerably improved the accuracy in breeding value estimates by accounting for both Mendelian sampling and historical coancestry that were not captured by the contemporary pedigree alone. Increasing family genotyping efforts were associated with continuous improvement in model fit, precision of genetic parameters, and breeding value accuracy. Yet, improvements were observed even at minimal genotyping effort, indicating that even modest genotyping effort is effective in improving genetic evaluation. The combined utilization of both pedigree and genomic information may be a cost-effective approach to increase the accuracy of breeding values in forest tree breeding programs where shallow pedigrees and large testing populations are the norm.

Maximizing genetic gain in tree breeding programs depends on the reliability of the estimated genetic parameters. Traits’ heritabilities and their genetic correlations are among the most critical genetic parameters determining potential genetic progress, therefore their precision is of great importance. Progeny testing is the vehicle by which these genetic parameters are estimated, while individual tree data, family composition, pedigree depth and connectedness, and field performance determine their reliability (Huber *et al.* 1992). Open-pollinated testing is often used to efficiently screen and assess large numbers of candidate trees (Burdon and Shelbourne 1971; Jayawickrama *et al.* 2000). However, it is recognized that treating open-pollinated families as “half-sibs” is inaccurate due to the effect of hidden relatedness, which cannot be easily unraveled in traditional pedigree-based analyses, thus affecting the resulting genetic parameters and candidate rankings (Squillace 1974; Askew and El-Kassaby 1994; Namkoong *et al.* 2012).

The use of molecular markers can uncover hidden relatedness and potential pedigree errors in open-pollinated populations via pedigree reconstruction and paternity assignment (Wang 2004; Kalinowski *et al.* 2007). Pedigree reconstruction has been effectively used to determine the genealogical relationship among groups of related individuals leading to estimating genetic parameters with increased precision (El-Kassaby and Lstibůrek 2009; Doerksen and Herbinger 2010; El-Kassaby *et al.* 2011; Koreckỳ *et al.* 2013; Klápště *et al.* 2014). Ritland (1996) proposed an innovative approach where marker-based relationship estimates could be used for estimating traits’ heritabilities and demonstrated the method’s utility in wild populations. Capitalizing on the availability of dense genomic marker sets, VanRaden (2008) derived estimates of marker-based relationships between pairs of individuals as a genomic relationship matrix (G), which can be used as a substitute to the traditional pedigree-based average numerator relationship matrix (A) in Henderson’s animal model (Henderson 1984; Frentiu *et al.* 2008; Habier *et al.* 2013; Klápště *et al.* 2014)

The advantages of using marker-based relationship estimates are: (1) bypassing the classical mating designs needed for generating structured pedigree (El-Kassaby *et al.* 2011; Klápště *et al.* 2014), (2) applicability to natural and experimental populations (El-Kassaby *et al.* 2012; Porth *et al.* 2013; Klápště *et al.* 2014), and (3) increased precision of genetic parameter estimates by accounting for genetic similarity due to common ancestry (*i.e.*, historical pedigree) (Powell *et al.* 2010) and Mendelian segregation (Visscher *et al.* 2006; Ødegård and Meuwissen 2012).

Combining the A with the marker-based relationship matrix G, in a single genetic covariance matrix H (Legarra *et al.* 2009; Misztal *et al.* 2009), has proven to be an effective way to improve relationship coefficients for better estimation of genetic parameters and tracking of genetic diversity in animal breeding systems. This method was dubbed “HBLUP,” since the best linear unbiased predictors (BLUPs) of breeding values are derived using the combined, H, genetic covariance matrix. The HBLUP method may be particularly useful in forest tree progeny test evaluation as the cost and logistics of genotyping the entirety of trials is prohibitive; thus, combining the marker-based relationship from a subset of trees from some families from few sites with the pedigree-based relationship estimator would be an efficient option for improving the derived genetic parameters’ precision for the entire test.

The aim of this study is to compare the traditional ABLUP approach to the HBLUP approach by comparison of the precision of genetic parameters, accuracy of breeding values, and rankings of selection candidates using variable proportions (0, 25, 50, 75, and 100%) of genotyped individuals within the open-pollinated families.

## Methods

### White spruce open-pollinated progeny test, phenotype data, and genotyping

Data for the present study was obtained from Beaulieu *et al.* (2014) and downloaded from the Dryad Digital Repository: doi 10.5061/dryad.6rd6f. Briefly, this study concerns a single white spruce [*Picea glauca* (Moench) Voss] provenance-progeny test site that was established in 1979 in Québec, Canada. The test site contains 214 open-pollinated families from 43 Québec provenances planted in randomly assigned five tree row plots at 2.4 m between row and 1.2 m within row spacing to attain a randomized block design with six blocks. A subset of 1694 trees were sampled from the site, the mean number of trees per family was ∼7.9, and the mean number of trees per family per block was ∼1.3. Complete details of the test site are available in Beaulieu *et al.* (2014). Phenotypic data for this study included 22 yr tree HT (cm) and WD (kg/m^3^) obtained via X-ray densitometry.

Single-nucleotide polymorphisms (SNPs) of the 1694 trees were genotyped using Illumina Infinium HD iSelect bead chip PgAS1 (Illumina, San Diego, CA) (Beaulieu *et al.* 2014). The SNPs were located within 2814 genes and separated by a minimum distance of 200 bp. Genes were selected from the white spruce gene catalog “GCAT” (Rigault *et al.* 2011) and were those of diverse functions (Pavy *et al.* 2013). A total of 6716 SNPs were available for use in the analyses.

### Data analyses

Traditional pedigree-based genetic analyses were performed under the assumptions of unrelated half-sibling family structure and a minimal population structure effect, as shown by Beaulieu *et al.* (2014). Variance components, heritabilities, genetic correlations, and individual tree breeding values were estimated with the bivariate animal model (herein referred to as “ABLUP”) (Henderson 1984) as follows:

$$y=\mathrm{X\beta}+Z_{1}a+Z_{2}u+e$$ (1)

where y is the response matrix of individually scaled and centered phenotypic observations for the two traits; X is the incidence matrix for the fixed effect β (trait means); $Z_{1}$ and $Z_{2}$ are the corresponding incidence matrices related to random additive genetic effects [breeding values, a∼N(0,G)] and block effects [u∼N(0,U)]; and the random residual error effects are distributed as e∼N(0,R). The covariance matrix for the random additive genetic effects was modeled using the heterogeneous covariance structure as $G=\left[\begin{array}{cc} \sigma_{a1}^{2} & \sigma_{a1a2}\\ \sigma_{a2a1} & \sigma_{a2}^{2} \end{array}\right]⊗A$, where A is the average numerator relationship matrix, $\sigma_{a1a2}$ is the additive covariance between traits 1 and 2, and ⊗ is the Kronecker product operator. The covariance matrix for the random block effects was modeled using diagonal structure as $U=\left[\begin{array}{cc} \sigma_{u1}^{2} & 0\\ 0 & \sigma_{u2}^{2} \end{array}\right]⊗I$ where I is an identity matrix. The random residual error effect was modeled using an unstructured covariance matrix structure as $R=\left[\begin{array}{cc} \sigma_{e1}^{2} & \sigma_{e1e2}\\ \sigma_{e2e1} & \sigma_{e2}^{2} \end{array}\right]⊗I$, where $\sigma_{e1e2}$ is the residual covariance between the two traits. Random effects were assumed to be independent.

The genomic relationship matrix (GRM) (G) was constructed after VanRaden (2008):

$$G=\mathrm{ZZ}\prime /2\sum_{j}p_{j}\left(1-p_{j}\right)$$ (2)

let n be the number of individuals and m be the number of markers, then Z is an n × m matrix of centered genotype scores with missing values replaced with zeros (*i.e.*, mean imputation), and $p_{j}$ is the reference allele frequency of the jth marker.

In the single-step model (herein referred to as “HBLUP”), the A matrix was substituted by the combined additive relationship matrix H (Legarra *et al.* 2009; Christensen and Lund 2010), resulting from accommodation of the genomic marker-based relationship matrix G:

$$H=\left[\begin{array}{cc} A_{11}+A_{12}A_{22}^{-1}\left(G-A_{22}\right)A_{22}^{-1}A_{21} & A_{12}A_{22}^{-1}G\\ GA_{22}^{-1}A_{21} & G \end{array}\right]$$ (3)

where $A_{11}$ is the block of A for the nongenotyped individuals of the population, ***A*_22_** is the block of genotyped individuals, and $A_{12}$ and $A_{21}$ are the blocks containing the expected additive genetic relationships between genotyped and nongenotyped individuals. The inverse of H ($H^{-1}$) can be obtained via (Aguilar *et al.* 2010; Christensen and Lund 2010):

$$H^{-1}=A^{-1}+\left[\begin{array}{cc} 0 & 0\\ 0 & G^{-1}-A_{22}^{-1} \end{array}\right]$$ (4)

where $A^{-1}$ is the inverse of the pedigree-based relationship matrix ***A***, and $G^{-1}$ and $A_{22}^{-1}$ are the pedigree and the genomic relationship matrices for the genotyped individuals, respectively.

Prior to calculation of H, the GRMs were scaled as G∗=βG+α, such that the average of diagonal and off-diagonal elements of ***G*** were equivalent to those of $A_{22}$ by solving the following system of equations for the parameters α and β (Christensen *et al.* 2012).

$$\{\begin{array}{c} \mathrm{Avg}\left(\mathrm{diag}\left(G\right)\right)\beta +\alpha =\mathrm{Avg}\left(\mathrm{diag}\left(A_{22}\right)\right)\\ \mathrm{Avg}\left(G\right)\beta +\alpha =\mathrm{Avg}\left(A_{22}\right) \end{array}$$ (5)

To avoid potential problems with inversion of G∗, it was weighted as (Aguilar *et al.* 2010):

$$G_{w}=0.95\times G*+\left(1-0.95\right)\times A_{22}$$ (6)

Five levels of genotyping effort were tested in the HBLUP method: 0, 25, 50, 75, and 100%. In the case of 0% of genotyping effort, variance components, genetic correlations, and breeding values were obtained strictly using expected additive genetic relationships defined in A (*i.e.*, ABLUP). At 100% genotyping effort, the G matrix used in Equation 4 containing all possible genotyped individuals was combined with the A matrix. In this last case, ***A***_11_ represents only the unrelated and nongenotyped maternal parents (*i.e.*, a diagonal square matrix of order 214). To avoid possible sampling bias, a replicated resampling scheme was used to obtain parameter estimates at 25–75% genotyping effort. In detail, two, four, or six individuals per family, of a possible maximum of eight, were randomly removed from the G and $A_{22}$ matrices prior to rescaling and combining with A in Equation 4. This process was repeated 30 times at each level of genotyping effort and mean values for the results are reported.

The restricted maximum likelihood (REML) was used to estimate variance and covariance for the random effects in the bivariate mixed model (1), and were obtained with the ASReml-R v3.0 program (Butler *et al.* 2009).

The single-trait narrow-sense heritability for of each trait from the bivariate ABLUP and HBLUP models were estimated as:

$${\hat{h}}^{2}=\frac{{\hat{\sigma }}_{a}^{2}}{{\hat{\sigma }}_{a}^{2}+{\hat{\sigma }}_{e}^{2}}$$ (7)

where ${\hat{\sigma }}_{a}^{2}$ and ${\hat{\sigma }}_{e}^{2}$ are the estimates of additive genetic and residual variances, respectively (Falconer and Mackay 1996).

The additive genetic correlation between the two traits was estimated as Pearson’s product moment:

$${\hat{r}}_{G}=\frac{{\hat{cov}}_{1,2}}{\sqrt{{\hat{\sigma }}_{a1}^{2}{\hat{\sigma }}_{a2}^{2}}}$$ (8)

where, ${\hat{cov}}_{1,2},$ ${\hat{\sigma }}_{a1}^{2},$ and ${\hat{\sigma }}_{a2}^{2}$ are the genetic covariance and additive genetic variances for traits 1 and 2 respectively, estimated from the bivariate model (1) (Falconer and Mackay 1996).

The theoretical accuracy of breeding values ($\hat{r}$) was estimated after (Dutkowski *et al.* 2002):

$$\hat{r}=\sqrt{1-\frac{SE_{i}^{2}}{\left(1+F_{i}\right){\hat{\sigma }}_{a}^{2}}}$$ (9)

where $SE_{i}$ is the standard error, and *F_i_* is the inbreeding coefficient of the *i*th individual. SEs of the breeding values were obtained from the bivariate model with variance components fixed to estimates from the same model with 100% genotyping effort for the respective traits, under the assumption that these were most accurate.

Additionally, the Akaike information criterion (AIC) was computed to compare the fit of the bivariate ABLUP and HBLUP models. A smaller AIC value indicates better fit. Furthermore, Spearman rank correlations and the proportion of common candidates in the top 5% of mothers and progenies were also calculated to compare whether the predicted breeding values differed among the ABLUP and HBLUP models.

### Data availability

The data used are available from the Dryad Digital Repository: doi:10.5061/dryad.6rd6f (Beaulieu *et al.* 2014). 

## Results

### Relationship matrices

Descriptive statistics for the expected (***A***) and combined (***H***) relationship estimators presented the ranges of the ***H*** relationship groups to be larger than that of ***A*** (Table 1). As expected, the Pearson correlation between ***A*** and the ***H*** relationship matrices decreased with an increase in genotyping effort. The ranges of the values within the various relationship groups also tended to expand with increasing genotyping effort. Particularly noteworthy was the presence of non-zero relationship coefficients between maternal parents given by the ***H*** relationship estimators, indicating that maternal parents are not completely unrelated as assumed in the pedigree. Likewise, large differences from the expected pairwise half-sib and unrelated values in ***A*** were observed in the ***H*** relationship estimators. These deviations imply the possible presence of pedigree errors on one side, and full-sibs within the half-sib families on the other side.

**Table 1:** Descriptive statistics of the pedigree-based (*A*) and combined pedigree marker-based (*H*) relationship estimators at 0, 25, 50, 75, and 100% genotyping effort

				Relationship Groups									
	Overall			Identity		Parent–Parent		Parent–Offspring		Half-Sib		Unrelated	
Estimator (GE) (%)	Mean	Variance	${\hat{r}}_{A}$	Mean (SD)	Range	Mean (SD)	Range	Mean (SD)	Range	Mean (SD)	Range	Mean (SD)	Range
***A*** (0)	0.002	0.001	1.000	1.000 (NA)	1.000–1.000	0.000 (NA)	0.000–0.000	0.500 (NA)	0.500–0.500	0.250 (NA)	0.250 – 0.250	0.000 (NA)	0.000–0.000
***H*** (25)	0.002*^a^*	0.001*^a^*	0.963*^a^*	0.996 (0.024)*^a^*	0.839–1.190*^a^*	0.000 (0.011)*^a^*	−0.031–0.219*^a^*	0.490 (0.032)*^a^*	0.362–0.667*^a^*	0.242 (0.031)*^a^*	−0.025–0.577*^a^*	0.001 (0.022)*^a^*	−0.076–0.661*^a^*
***H*** (50)	0.002*^a^*	0.001*^a^*	0.890*^a^*	0.990 (0.044)*^a^*	0.752–1.292*^a^*	0.000 (0.017)*^a^*	−0.050–0.362*^a^*	0.468 (0.071)*^a^*	0.264–0.792*^a^*	0.226 (0.068)*^a^*	−0.032–0.668*^a^*	0.001 (0.024)*^a^*	−0.084–0.791*^a^*
***H*** (75)	0.002*^a^*	0.001*^a^*	0.816*^a^*	0.989 (0.057)*^a^*	0.669–1.380*^a^*	0.000 (0.022)*^a^*	−0.061–0.457*^a^*	0.452 (0.098)*^a^*	0.203–0.860*^a^*	0.213 (0.098)*^a^*	−0.033–0.712*^a^*	0.001 (0.027)*^a^*	−0.086–0.860*^a^*
***H*** (100)	0.002	0.001	0.754	0.990 (0.065)	0.599–1.424	0.000 (0.025)	−0.068–0.574	0.442 (0.117)	0.169–0.894	0.204 (0.122)	−0.033–0.754	0.001 (0.031)	−0.087–0.981

Sib, sibling; GE, Genotyping effort; ${\hat{r}}_{A}$, Pearson product moment correlation with pedigree relationship estimator ***A***; ***A***, pedigree-based relationship estimator; ***H***, combined pedigree marker-based relationship estimator; NA, not applicable.

a Mean value(s).

Family and provenance structure was visually evident in the heat maps of the relationship estimators (see Supplemental Material, Figure S1, Figure S2, Figure S3, Figure S4, Figure S5 in File S1). Samples were sorted according to family and provenance, then the pairwise relationship coefficients of the matrices were plotted. Historical relationships (*i.e.*, provenance) not captured by the pedigree estimator ***A*** (Figure S1) were visually apparent in ***H*** with as little as 25% genotyping effort (Figure S2). Further, potential pedigree errors not accounted for in the contemporary pedigree were uncovered, these errors may be due to mislabeling or technical errors in the lab. Population structure and potential pedigree errors were further evidenced by the large deviations observed between the ranges maternal parent–parent, half-sib, and unrelated relationship coefficients between ***A*** and the ***H*** relationship estimators (Table 1).

### Variance components

The additive genetic variance estimates obtained from the bivariate HBLUP models were substantially lower than the bivariate ABLUP model estimates for both HT and WD (Table 2). Further, the additive genetic component estimates of the HBLUP models continually decreased as a result of increased genotyping effort. It should be noted that the observed differences in additive genetic variance estimates between the ABLUP and HBLUP models reflects on the known bias associated with considering wind-pollinated offspring as half-sib progeny, which often results in the overestimation of additive genetic variance and subsequently incorrect parental and progeny rankings. This bias is attributable to hidden relationships in assumed open-pollinated half-sib pedigrees.

**Table 2:** Pedigree-based (*A*) and combined pedigree marker-based (*H*) estimates of variance components for white spruce tree height and wood density at 0, 25, 50, 75, and 100% genotyping effort

Estimator (GE) (%)	${\hat{\sigma }}_{a}^{2}$ (HT)	${\hat{\sigma }}_{b}^{2}$ (HT)	${\hat{\sigma }}_{e}^{2}$ (HT)	${\hat{h}}^{2}$ (HT)	${\hat{\sigma }}_{a}^{2}$ (WD)	${\hat{\sigma }}_{b}^{2}$ (WD)	${\hat{\sigma }}_{e}^{2}$ (WD)	${\hat{h}}^{2}$ (WD)	${\hat{r}}_{G}$	AIC
***A*** (0%)	0.284 (0.074)	0.046 (0.031)	0.679 (0.071)	0.295 (0.074)	0.589 (0.100)	0.010 (0.008)	0.403 (0.086)	0.594 (0.091)	−0.035 (0.148)	3125.586
***H*** (25%)	0.271 (0.067)*^a^*	0.046 (0.031)*^a^*	0.692 (0.064)*^a^*	0.282 (0.067)*^a^*	0.487 (0.080)*^a^*	0.010 (0.008)*^a^*	0.486 (0.0691)*^a^*	0.502 (0.075)*^a^*	−0.043 (0.141)*^a^*	2907.071*^a^*
***H*** (50%)	0.228 (0.055)*^a^*	0.046 (0.031)*^a^*	0.731 (0.054)*^a^*	0.237 (0.055)*^a^*	0.403 (0.064)*^a^*	0.009 (0.008)*^a^*	0.567 (0.055)*^a^*	0.416 (0.060)*^a^*	−0.050 (0.138)*^a^*	2672.915*^a^*
***H*** (75%)	0.196 (0.046)*^a^*	0.046 (0.031)*^a^*	0.762 (0.047)*^a^*	0.205 (0.046)*^a^*	0.339 (0.052)*^a^*	0.009 (0.008)*^a^*	0.628 (0.046)*^a^*	0.351 (0.049)*^a^*	−0.043 (0.134)*^a^*	2379.521*^a^*
***H*** (100%)	0.170 (0.039)	0.045 (0.031)	0.786 (0.042)	0.178 (0.039)	0.300 (0.046)	0.009 (0.008)	0.666 (0.041)	0.310 (0.043)	−0.023 (0.133)	1989.502

${\hat{\sigma }}_{a}^{2}$, ${\hat{\sigma }}_{b}^{2}$, ${\hat{\sigma }}_{e}^{2}$ are the estimated additive genetic, block, and residual variance components. SEs in parentheses. HT, height in cm; ${\hat{h}}^{2}$estimated narrow-sense individual tree heritability; WD, wood density in kg/m^3^; ${\hat{r}}_{G}$, additive genetic correlation; AIC, Akaike information criterion model fit statistic; ***A***, pedigree-based; ***H***, combined pedigree marker-based.

a Mean value(s).

Narrow-sense heritability estimates of the bivariate HBLUP models mirrored those of the additive genetic variance estimates (HT: from 0.178 to 0.295; WD: from 0.310 to 0.594). These estimates at 100% genotyping effort for HT and WD were intermediate to the polygenic and marker-based estimates described by Beaulieu *et al.* (2014). The trend observed in the estimates of additive genetic variance was coupled with an increase in the residual variance component for the HBLUP models compared to the ABLUP model, with the lowest estimates observed for the ABLUP model, indicating that some residual variance was shifted to the additive variance, hence the overestimation of heritability (Table 2). Comparison of the block variance estimates provided no pattern of association with any factor, and the estimates were relatively stable across all models for both WD and HT. Across all the models, the additive genetic correlations between HT and WD were minor, negative, and not significant (based on SEs). The variation in model parameters’ estimates under 25, 50, and 75% genotyping effort, due to random sampling, was low, indicating that the selection of genotyped individuals within family has minimal impact (see Table S1, Table S2, and Table S3 in File S1).

Large incremental improvements in the model fit, determined by AIC, were observed for the HBLUP models over the ABLUP model with increasing genotyping effort. Model fit was optimal in the case with complete genotyping effort (100%), using the ***H*** relationship estimator (Table 2).

### Breeding value accuracy

The mean breeding value accuracies for HT and WD are presented for three classes of individuals: maternal parents and genotyped and nongenotyped progeny (Table 3). Comparison of ***H*** and ***A*** relationship estimators on mean breeding value accuracies for the maternal parents showed minor increases for HT (differences were limited to only the second decimal place; from 0.520 to 0.539) and no changes for WD (from 0.635 to 0.635) with increasing family genotyping efforts. The mean breeding value accuracy of nongenotyped progeny using the ***H*** estimator across all levels of genotyping effort was stable and equivalent to the mean breeding value accuracy of the progeny using the ***A*** estimator, for both HT and WD. As expected, the largest improvements over the ***A*** relationship estimator were noted for genotyped progeny using the ***H*** estimators. Increasing genotyping effort produced large increases in mean breeding value accuracy for both HT and WD over mean breeding value accuracies of the progeny using ***A*** (HT: 0.474; WD: 0.605), with the effect greater for HT (HT: from 0.498 to 0.536; WD: from 0.626 to 0.661).

**Table 3:** Mean breeding value accuracy estimates ($\hat{r}$) for white spruce tree height and wood density using pedigree-based (*A*) and combined pedigree marker-based (*H*) relationship estimators at 0, 25, 50, 75, and 100% genotyping effort for three classes of individuals: maternal parent, genotyped progeny, and nongenotyped progeny

Estimator (GE) (%)	Class	HT	WD
		$\hat{r}$	$\hat{r}$
***A*** (0%)	Maternal	0.521 (NA)	0.635 (NA)
	Genotyped	NA	NA
	Nongenotyped	0.474 (NA)	0.605 (NA)
***H*** (25%)	Maternal	0.525 (0.0007)	0.635 (0.0007)
	Genotyped	0.499 (0.0008)	0.626 (0.0007)
	Nongenotyped	0.474 (0.0003)	0.604 (0.0002)
***H*** (50%)	Maternal	0.527 (0.0010)	0.635 (0.0009)
	Genotyped	0.514 (0.0006)	0.640 (0.0004)
	Nongenotyped	0.473 (0.0004)	0.602 (0.0003)
***H*** (75%)	Maternal	0.530 (0.0007)	0.635 (0.0007)
	Genotyped	0.528 (0.0004)	0.651 (0.0003)
	Nongenotyped	0.475 (0.0003)	0.601 (0.0002)
***H*** (100%)	Maternal	0.539 (NA)	0.635 (NA)
	Genotyped	0.536 (NA)	0.661 (NA)
	Nongenotyped	NA	NA

SD of sampling distribution in parentheses. HT, height in cm; WD, wood density in kg/m^3^; $\hat{r}$,breeding value accuracy estimates; ***A***, pedigree-based relationship estimator; NA, not applicable; ***H***, combined pedigree marker-based relationship estimator.

### Candidate rankings

The Spearman rank correlations for maternal and progeny breeding values, and the proportion of common candidates in the top 5% of maternal and progeny rankings, between the ***A*** and ***H*** relationship estimator at 25, 50, 75, and 100% genotyping effort, for both HT and WD, are presented in Table 4. The pairwise Spearman rank correlations were always less than perfect indicating at least some disagreement in the rankings of candidate maternal parents and progeny. Overall, maternal and progeny Spearman rank correlations between the HBLUP models and the ABLUP model were greater for WD than HT, further suggesting that genomic information was less impactful on candidate rankings for the higher heritability trait. Furthermore, the Spearman rank correlations between the ABLUP model and the HBLUP models decreased with increasing genotyping effort in all cases. The top 5% analysis again showed overall better agreement for WD over HT for both maternal and progeny candidates. Furthermore, agreement with the top 5% ABLUP candidates decreased with increasing genotyping effort in the HBLUP models. In summary, the Spearman rank correlations and the proportion of common candidates in the top 5% suggests that the inclusion of genomic information affects candidate rankings and has the potential to alter both forward and backward selections.

**Table 4:** Proportion of common candidates in the top 5% of maternal and progeny rankings (above diagonals), and estimated Spearman breeding value rank correlations (diagonals and below diagonals), for pedigree-based (*A*) and combined pedigree marker-based (*H*) relationship estimators at 0, 25, 50, 75, and 100% genotyping effort for white spruce tree height and wood density

	Parent						Progeny				
	***A*** (0%)	***H*** (25%)	***H*** (50%)	***H*** (75%)	***H*** (100%)		***A*** (0%)	***H*** (25%)	***H*** (50%)	***H*** (75%)	***H*** (100%)
HT											
* ****A*** (0%)	**1.00 (NA)**	0.89 (0.052)*^a^*	0.78 (0.070)*^a^*	0.70 (0.055)*^a^*	0.64 (NA)	***A*** (0%)	**1.00 (NA)**	0.89 (0.035)*^a^*	0.76 (0.040)*^a^*	0.66 (0.034)*^a^*	0.55 (NA)*^a^*
* ****H*** (25%)	0.99 (0.002)*^a^*	**0.98 (0.002)***^a^*	0.82 (0.046)*^a^*	0.74 (0.045)*^a^*	0.69 (0.045)*^a^*	***H*** (25%)	0.98 (0.003)*^a^*	**0.98 (0.002)***^a^*	0.74 (0.012)*^a^*	0.66 (0.015)*^a^*	0.56 (0.023)*^a^*
* ****H*** (50%)	0.96 (0.006)*^a^*	0.97 (0.002)*^a^*	**0.97 (0.004)***^a^*	0.80 (0.049)*^a^*	0.78 (0.062)*^a^*	***H*** (50%)	0.94 (0.010)*^a^*	0.95 (0.002)*^a^*	**0.94 (0.006)***^a^*	0.70 (0.017)*^a^*	0.64 (0.034)*^a^*
* ****H*** (75%)	0.98 (0.006)*^a^*	0.95 (0.003)*^a^*	0.97 (0.005)*^a^*	**0.98 (0.002)***^a^*	0.88 (0.065)*^a^*	***H*** (75%)	0.90 (0.008)*^a^*	0.91 (0.003)*^a^*	0.92 (0.006)*^a^*	**0.94 (0.002)***^a^*	0.77 (0.024)*^a^*
* ****H*** (100%)	0.91 (NA)	0.93 (0.004)*^a^*	0.96 (0.006)*^a^*	0.98 (0.002)*^a^*	**1.00 (NA)**	***H*** (100%)	0.84 (NA)	0.86 (0.004)*^a^*	0.90 (0.006)*^a^*	0.95 (0.004)*^a^*	**1.00 (NA)***^a^*
WD											
* ****A*** (0%)	**1.00 (NA)**	0.90 (0.058)*^a^*	0.77 (0.066)*^a^*	0.7 (0.050)*^a^*	0.73 (NA)	***A*** (0%)	**1.00 (NA)**	0.92 (0.018)*^a^*	0.83 (0.023)*^a^*	0.74 (0.018)*^a^*	0.70 (NA)
* ****H*** (25%)	1.00 (0.001)*^a^*	**0.99 (0.001)***^a^*	0.81 (0.031)*^a^*	0.76 (0.051)*^a^*	0.79 (0.060)*^a^*	***H*** (25%)	0.99 (0.002)*^a^*	**0.99 (0.001)***^a^*	0.87 (0.014)*^a^*	0.80 (0.015)*^a^*	0.76 (0.017)
* ****H*** (50%)	0.98 (0.003)*^a^*	0.98 (0.001)*^a^*	**0.98 (0.002)***^a^*	0.83 (0.052)*^a^*	0.85 (0.070)*^a^*	***H*** (50%)	0.96 (0.005)*^a^*	0.97 (0.001)*^a^*	**0.96 (0.002)***^a^*	0.85 (0.012)*^a^*	0.84 (0.023)
* ****H*** (75%)	0.96 (0.005)*^a^*	0.97 (0.002)*^a^*	0.98 (0.003)*^a^*	**0.98 (0.001)***^a^*	0.94 (0.059)*^a^*	***H*** (75%)	0.92 (0.006)*^a^*	0.93 (0.002)*^a^*	0.94 (0.003)*^a^*	**0.96 (0.001)***^a^*	0.91 (0.019)
* ****H*** (100%)	0.95 (NA)	0.96 (0.002)*^a^*	0.97 (0.004)*^a^*	0.99 (0.002)*^a^*	**1.00 (NA)**	***H*** (100%)	0.88 (NA)	0.90 (0.003)*^a^*	0.92 (0.004)*^a^*	0.96 (0.003)*^a^*	**1.00 (NA)**

SD of sampling distribution in parentheses. ***A***, pedigree-based relationship estimator; ***H***, combined pedigree marker-based relationship estimator; HT, height in cm; NA, not applicable; WD, wood density in kg/m^3^.

a Mean value(s).

## Discussion

The investigation of genetic architecture in forest trees requires the establishment of large scale testing populations with predefined mating design. The results’ quality (*i.e.*, accuracy and precision of genetic parameters) are often a reflection of the extent of efforts invested into the design of the field experiment. Commonly, quantitative genetics analyses are based on the animal model implemented in mixed model theory (Henderson 1984). Such analyses are based on the average numerator relationship matrix tracking probability of identity by descent (***A*** matrix). The mixed model theory operates with the assumption that variance–covariance matrices of random terms used in the animal model are error free (Henderson 1984). The average numerator relationship matrix cannot fulfill this assumption as it is based on the pairwise expected relationship values and does not account for the Mendelian sampling term. Moreover, the contemporary shallow pedigrees of many forest tree breeding programs are unable to account for historical coancestry. The use of genotypic data enables greater insight into genetic covariances and the precise mapping of the Mendelian sampling term (Visscher *et al.* 2006). The use of marker-based relationship matrix analyses can be beneficial even in small populations with limited pedigree information (El-Kassaby *et al.* 2012; Ødegård and Meuwissen 2012; Porth *et al.* 2013; Cappa *et al.* 2016)

However, the cost and logistics of obtaining genotypic information for the large testing populations in tree improvement programs still represents a barrier to widespread implementation of this methodology (Beaulieu *et al.* 2014). Additionally, deployment of genetic analysis using the entire testing population (*i.e.*, genotyped and nongenotyped individuals) is important to eliminate bias connected to the total reliance on using genotyped individuals only or error-prone multi-step methods. While the genomic information benefits from lower inbreeding build-up during the selection phases as compared to pedigree-based BLUP (Liu *et al.* 2014) due to the added knowledge about the Mendelian sampling term and historical coancestry, it is recommended that including nongenotyped individuals in the same data analysis is beneficial as it effectively reduces the bias associated with selection of individuals for genotyping (Ducrocq and Patry 2010). This is increasingly important, especially in forest tree breeding programs, which are based on shallow pedigree selection from large base populations. The HBLUP method combining both pedigree- and marker-based relationship matrices generally provides as good as, or better, results over pedigree-based BLUP (Christensen *et al.* 2012) or multi-step approaches (Vitezica *et al.* 2011).

By permitting both pedigree and marker information in a single genetic evaluation analysis, the combined HBLUP approach offers resolution to these problems. Information gained through genomic relationship estimates for a subset of genotyped individuals is reflected via pedigree relationships to the nongenotyped individuals in the relationship matrix ***H*** (Legarra *et al.* 2009). Thus, more accurate estimates of relatedness are obtained in a cost-efficient manner. Our results support this notion through large improvement in the model fit as represented by the AIC statistics from the exclusive pedigree-based model to models utilizing the combination of marker and pedigree information at various genotyping efforts (Table 2). Furthermore, the presence of hidden relatedness, particularly in open-pollinated trials, can be a serious problem in their genetic evaluation resulting in upwardly biased estimates of heritability (Squillace 1974; Askew and El-Kassaby 1994; Namkoong *et al.* 2012) (Table 2). This bias is caused by overestimation of additive genetic variance due to the unrealistic assumption of pure half-sibling relatedness within the open-pollinated families, as well as complete unrelatedness among the parental donors. Reality and empirical evidence suggests the existence of hidden relationships within these families (*i.e.*, full-sibs, half-selfs, and full-selfs) (Squillace 1974; Askew and El-Kassaby 1994; Namkoong *et al.* 2012; Gamal El-Dien *et al.* 2016). Our results confirm that hidden relatedness within the OP families and historical coancestry exists in this *P. glauca* population, and was better accounted for through the HBLUP method, with increasing genotyping effort helping to approach more realistic estimates of genetic parameters and better model fit as compared to the analysis performed on the complete pedigree alone (Table 2). It should be stated that all hidden relatedness can only be accounted for under the GBLUP analysis, where individuals are genotyped (including parents); however, the HBLUP is composed of a mixture of genotyped and nongenotyped individuals, thus a slight overestimation of the additive genetic variance is expected. The degree of additive variance overestimation should lie between the GBLUP and pedigree analyses (Beaulieu *et al.* 2014).

The accuracy of the estimated breeding values is of practical importance to tree breeders. Correct ranking of the selection candidates based on accurate estimates of relatedness is an important factor. While traditional pedigree-based analyses have been proven to deliver increased genetic gain, a reduction in the size of the testing population can be achieved with improvement in accuracy through the use of genetic markers in the evaluation. The comparison of breeding value accuracies in this study was based on the assumption that the variance component estimates produced by the bivariate HBLUP model with full genotyping effort were most accurate. We fixed the variance components of each model to these estimates prior to calculating theoretical breeding value accuracy for each scenario. Our results show incremental improvement in the accuracy of progeny breeding values for the genotyped subset for both HT and WD with increasing genotyping effort (Table 3). However, this improvement was not translated to the nongenotyped progeny subset in either trait. The simple, disconnected pedigree structure of this population is a probable explanation for this observation, as the genomic information is only transferred from genotyped to nongenotyped individuals via pedigree relationships. We expect that in systems with more complex and interconnected pedigree structure, such as diallel mating designs, multi-generational complex pedigrees will benefit more from the HBLUP method as genomic information can be inferred from multiple family sources to the nongenotyped individuals.

Furthermore, family or maternal parent breeding value accuracy remained constant across all levels of genotyping effort for the trait with greater heritability, WD, whereas slight increases were observed for the lower heritability trait, HT, at 25, 50, 75, and 100% genotyping effort (from 0.525 to 0.539). The difference in contrasting heritabilities of the traits and these findings agree with previous discussions that genomic selection is expected to be more efficient in low heritability traits (Calus *et al.* 2008; Grattapaglia and Resende 2011). Yet, the inclusion of even a small proportion (*i.e.*, 25%) of genomic information per family produced differences in ranking the top 5% of both maternal and progeny candidates for both traits (Table 4).

The accuracy of estimated breeding values in HBLUP is not a function of only average diagonal and off-diagonal elements in the ***G*** matrix, but also the difference between them as it reflects the level of relatedness in the testing population (Forni *et al.* 2011). Generally, the genomic relationship matrix uses actual allele frequencies instead of those of the base population, which are usually unknown (in this case, the base population is treated as the studied pedigree population), and sets the genomic-base population as the genotyped population (Oliehoek *et al.* 2006). However, such analysis is affected by incompatibility between pedigree- and marker-based relationship matrices due to the inferences made about the base population using information from the studied population. Powell *et al.* (2010) highlighted the importance of scaling these matrices by correcting the genomic relationship matrix by a factor reflecting the mean difference between the ***A*** and ***G*** matrices. Several approaches were proposed to rescale the ***G*** matrix according to the ***A*** matrix (Meuwissen *et al.* 2011; Vitezica *et al.* 2011; Christensen *et al.* 2012); however, an opposite approach was also proposed by Christensen *et al.* (2012), whereby the ***A*** matrix is rescaled according to its ***G*** counterpart. To avoid incompatibility between the ***G*** and ***A*** matrices, a normalized ***G*** matrix containing the average of diagonal elements equal to one needs to be constructed. Such a matrix is efficient when the population is inbreeding free, and if inbreeding is present then inbreeding coefficients have to be included in the denominator (Forni *et al.* 2011). We chose to scale ***G*** according to Christensen *et al.* (2012); however, all analyses were tested without scaling ***G*** and yielded comparable results (results not shown). This result is likely due to the shallow pedigree structure and minor differences between the ***A***_22_ and ***G*** matrices. Additionally, Vitezica *et al.* (2011) observed discrepancies in the accurate genomic breeding values with incorrectly scaled ***G***, but only under strong selection (*i.e.*, nonrandom) of genotyped individuals and when genotyping was across 10 generations. However, in the present study, the 25–75% of genotyped trees were randomly removed (*i.e.*, unselected) and all these trees came from only one generation.

As a preliminary investigation into the combined use of genomic and pedigree information in the applied genetic analysis of white spruce, the HBLUP method has proven to be a beneficial tool to forest tree breeders. The inclusion of nongenotyped and genotyped trees in a single analysis produced improvements in breeding value accuracy and model fit, particularly for the trait with low heritability (HT). Further, discordance in candidate rankings between the HBLUP and the traditional method that utilizes ***A*** were observed. Improvement in the results were continuous with increasing genotyping effort; however, improvements were also seen at the minimum level of 25% genotyping effort.

## Supplementary Material

Supplemental material is available online at www.g3journal.org/lookup/suppl/doi:10.1534/g3.116.037895/-/DC1.
