# Supplementary material for: Single-Step BLUP with Varying Genotyping Effort in Open-Pollinated Picea glauca
Source: G3 (Bethesda). 2017 Jan 24;7(3):935–42. doi: 10.1534/g3.116.037895 (PMC5345723; doi:10.1534/g3.116.037895)

Fig. S1. The procedure leading to the assessment of the selection strength, at the amino acid level, on synonymous codons usage (the orange box). To achieve that, a mutation-selection model (the green boxes) was developed. It results from connecting the selection on amino acid substitutions (the blue box) with a codon substitution process derived from a nucleotide mutation process (the yellow boxes). To find the minimum and maximum of this selection strength, nucleotide rate matrices were optimized in Evolutionary Strategies approach (the pink box).

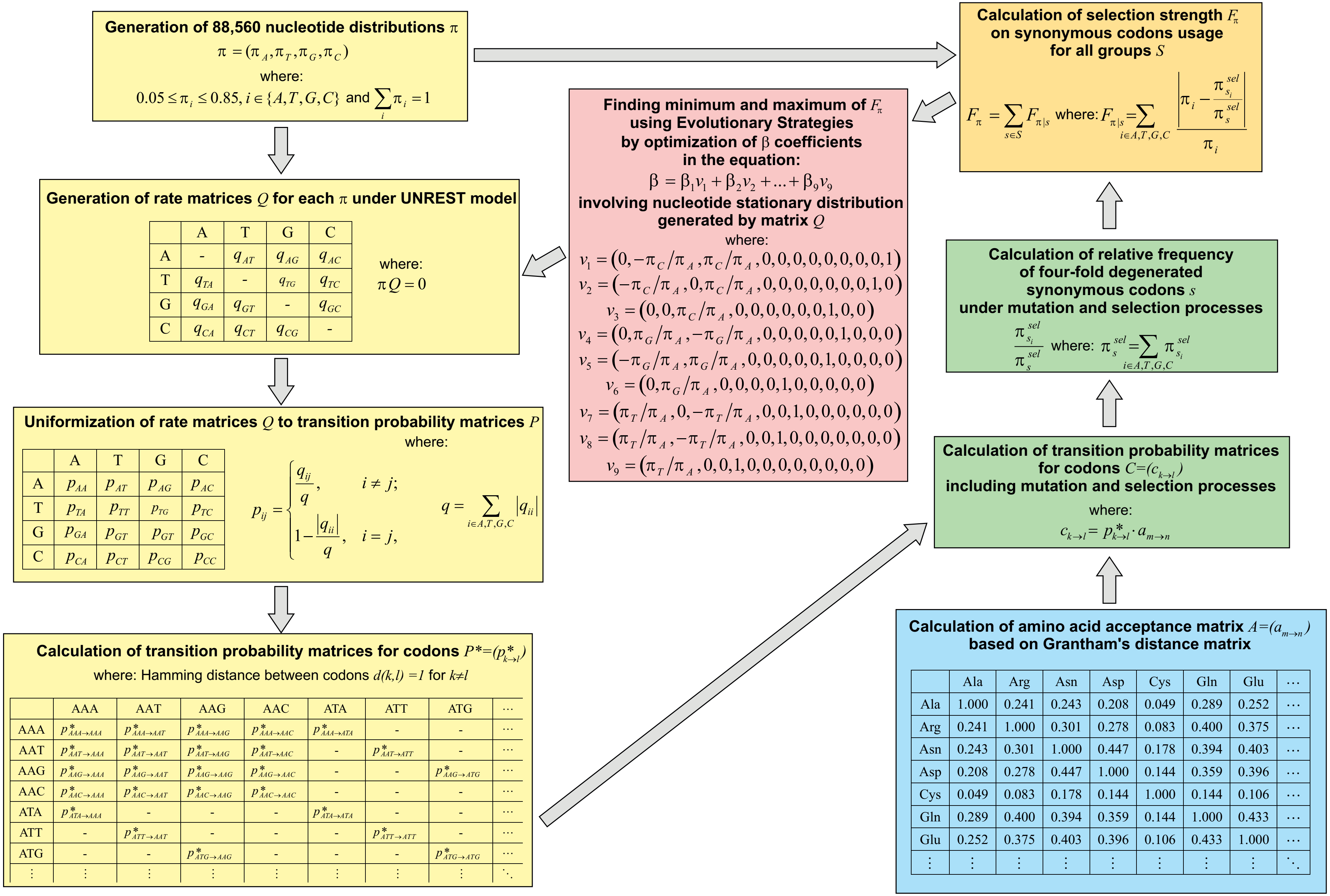

Supplement: Supplementary file 1 [file 935FigureS1.pdf]
