# Supplementary material for: Single-Step BLUP with Varying Genotyping Effort in Open-Pollinated Picea glauca
Source: G3 (Bethesda). 2017 Jan 24;7(3):935–42. doi: 10.1534/g3.116.037895 (PMC5345723; doi:10.1534/g3.116.037895)

Fig. S2. Comparison of nucleotide substitution probabilities for 5% of top matrices that maximized the values of F_{π} (the normalized difference between the relative frequency of four-fold degenerated codons after the selection on amino acids and their expected frequency resulting only from a mutation process). The thick line indicates median, the grey box shows quartile range and the whiskers determine the range without outliers.

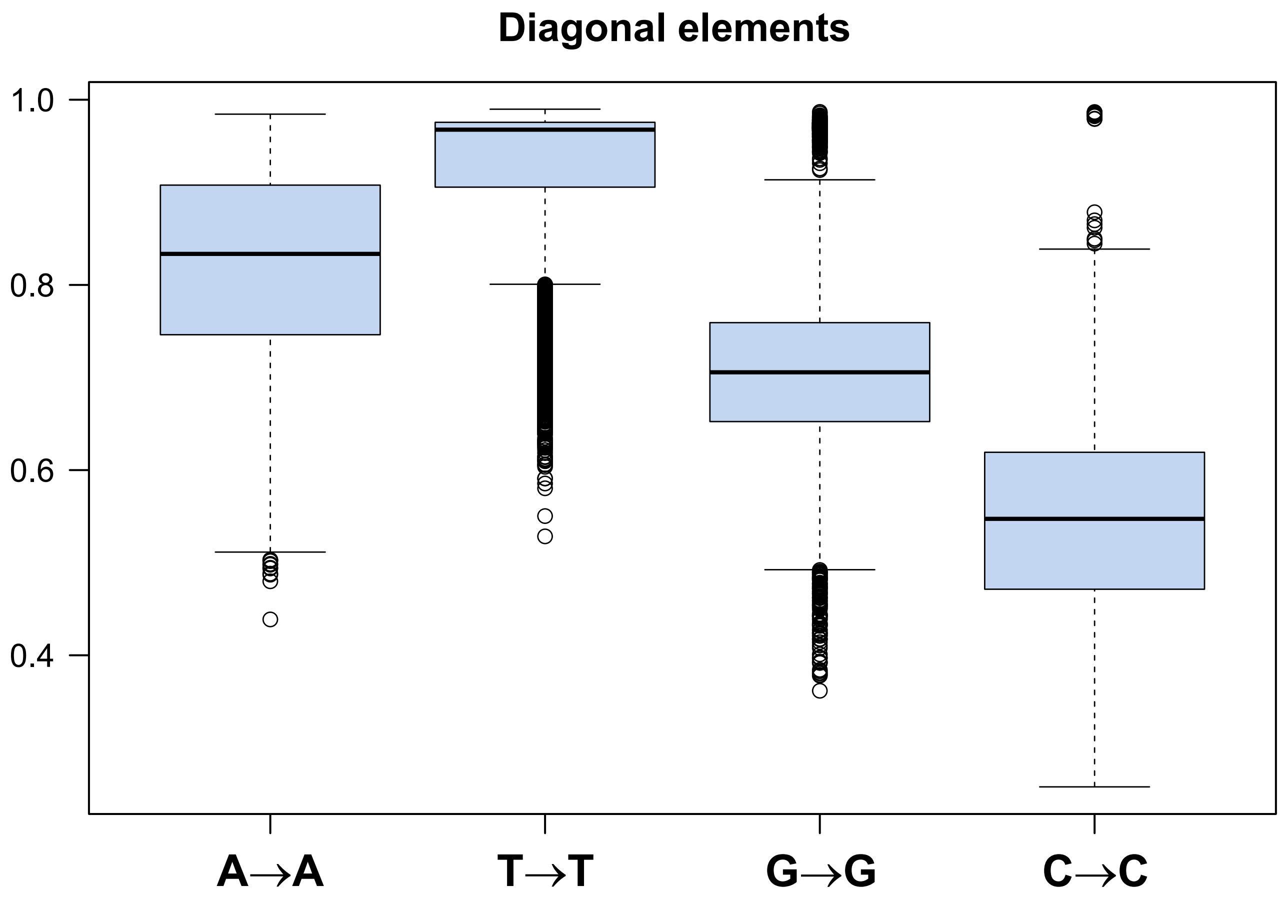

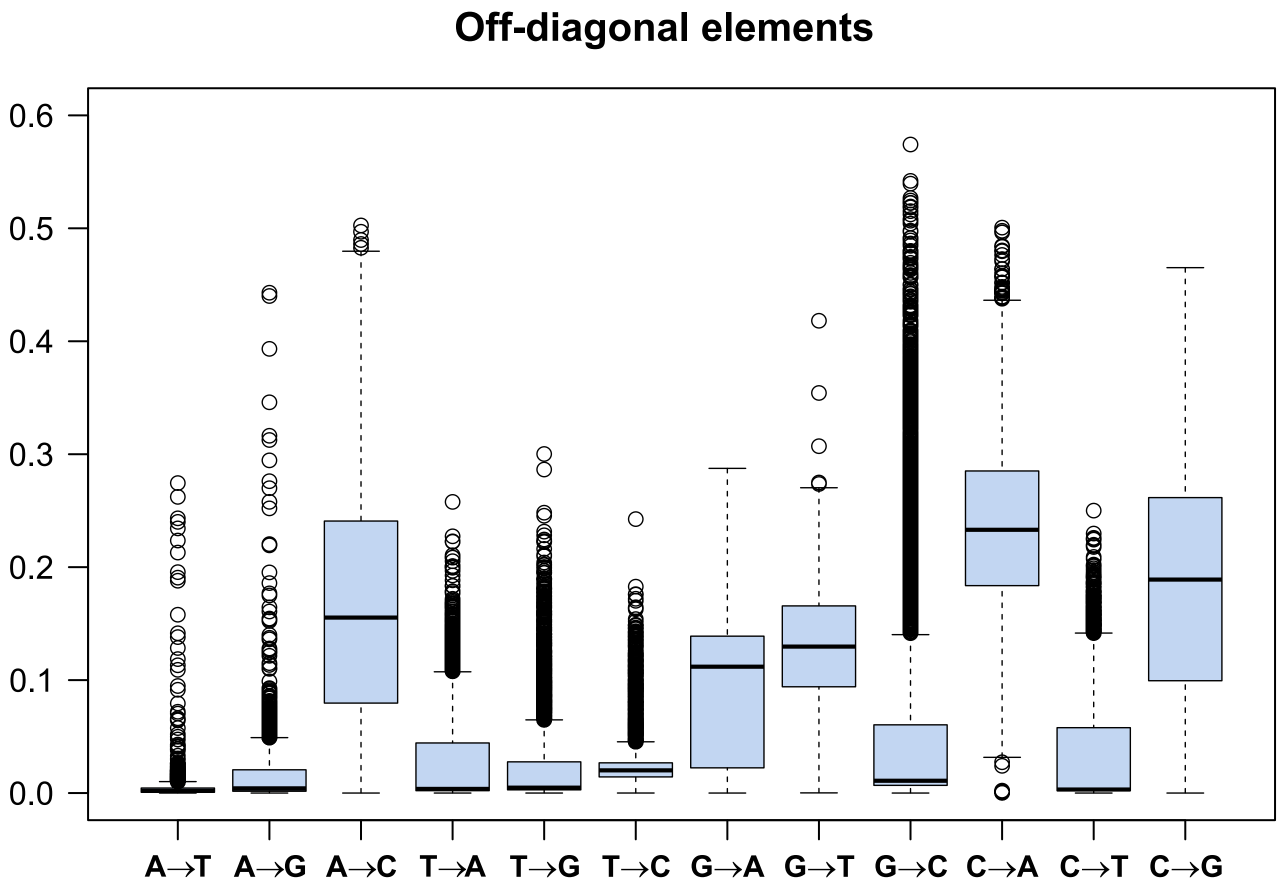

Supplement: Supplementary file 2 [file 935FigureS2.pdf]
